# Development and Evaluation of a German Suicide Prevention Website for Men: Exploratory Study

**DOI:** 10.2196/84733

**Published:** 2026-04-06

**Authors:** Cora Spahn, Jan-Gabriel Dobroschke, Lena Spangenberg, Heide Glaesmer

**Affiliations:** 1 Department of Medical Psychology and Medical Sociology, Leipzig University, Philipp-Rosenthal-Strasse 55, Leipzig, 04103, Germany, 49 3419718801, 49 3419718809

**Keywords:** suicide, men, prevention, e-mental health, evaluation

## Abstract

**Background:**

Men face a substantially higher risk of suicide. Effective suicide prevention strategies for men should specifically target gender-related risk factors, such as their lower likelihood of seeking professional help.

**Objective:**

This study investigates the use and impact of a suicide prevention website for men between March 1, 2023, and December 31, 2024. The Männer Stärken website is the first suicide prevention platform for men in Germany, with the primary aim of facilitating help-seeking behavior. The development of the platform was informed by interviews with men who had attempted suicide, as well as by existing evidence on effective communication strategies for engaging men at risk.

**Methods:**

This exploratory study combines quantitative web analytics and survey data with a qualitative analysis of open-ended responses from a feedback form. Using the web analytics tool Matomo, data were collected on the number of visits to the website and the subpages they accessed. In addition, 291 anonymous feedback forms were analyzed regarding visitors’ perceptions of the website’s helpfulness and its potential to support help-seeking behavior. A further component involved an online survey (n=40) examining whether a short suicide prevention film featured on the website could increase the intention to seek help.

**Results:**

During the study period, the website recorded 29,279 visits. A majority (n=291) of the respondents reported via the feedback form that they found the website helpful (n=201, 69.1%) and believed it could encourage help-seeking behavior (59.8%). In the evaluation of the short film, a significant increase in participants’ intentions to seek help was observed in situations involving suicidal ideation and personal difficulties and when considering professional support services. This effect was not observed with regard to informal sources of support, such as friends or family.

**Conclusions:**

The data suggest that the website is being used. Among those who completed the anonymous survey form (N=291), a majority reported that the website fulfills its primary aim of providing helpful pathways to support services. The evaluation of the short film further supports this conclusion. However, certain limitations must be acknowledged: since the data were collected in a field setting, the ability to draw firm conclusions about the characteristics or representativeness of the visitor sample is limited. In addition, the sample size for the short film evaluation was small. Nevertheless, the findings point to a clear need for gender-specific suicide prevention initiatives. They indicate promising directions for the development of effective, low-threshold measures, which merit further investigation in future research.

## Introduction

Worldwide, men are at significantly greater risk of dying by suicide. In Germany, men are approximately 3 times more likely to die by suicide than women [1]. Various risk factors for suicide in men are discussed in connection with this markedly increased risk. In a review of gender-specific risk factors for suicidal behavior, Richardson et al [2] found the strongest evidence for diagnosed depression, alcohol and/or drug use or dependence, and being single or widowed. Men also tend to use more lethal methods when attempting suicide [3-5]. Another important risk factor is that men are less likely to seek psychosocial help during suicidal crises and are less inclined to disclose suicidal thoughts within the support system [6-8]. Studies indicate that stigma and masculinity norms that discourage help-seeking (eg, self-reliance) may constitute barriers to seeking and accessing support [9-11].

The significantly higher suicide risk among men indicates that they are an important target group for suicide prevention. However, men are less likely than women to be reached by general, nongender-specific suicide prevention efforts [12]. To date, only a few suicide prevention programs worldwide specifically address men and their gender-specific risk factors. Promising examples from Australia include the MOVEMBER men’s health promotion initiative [13] and the 2016 suicide prevention campaign MAN-UP [14-16]. The MAN-UP program consists of a 3-part documentary and a related website [17], both of which focus on masculinity, mental health or suicidality, and help-seeking. King et al [15,18] demonstrated that the website is used as a gateway to access support organizations and helps men to engage with the MAN UP team on topics such as suicidality and mental health. In addition, there is a Belgian suicide prevention campaign, “Get Out of Your Head,” which also specifically targets men and includes information and testimonials from men with lived experience. A Dutch-language (Flemish) website [19] was developed and evaluated in 2022. Stas et al [20] demonstrated that the website led to an increase in help-seeking intentions and greater openness to communicating emotions among men.

In March 2023, the first German-language website dedicated to suicide prevention among men was launched [17]. The website was developed as part of the MEN-ACCESS project. In addition to the website for men at risk of suicide, an online program for gatekeepers was developed as part of the MEN-ACCESS project [21], and both measures were initially evaluated through qualitative interviews [22]. In Germany, the German Society for Suicide Prevention (Deutsche Gesellschaft für Suizidprävention) provides an online overview of resources for individuals with lived experiences and professionals [23]. The website is briefly presented and linked to as a dedicated resource for men in suicidal crises.

The website incorporates aspects and techniques that have been shown to improve help-seeking behavior among men [24]. These include the use of role models to deliver information, psychoeducation to enhance knowledge about mental health as well as problem-solving strategies, support in identifying symptoms, motivation for behavioral change, the introduction of and information about available support services, and the positive reframing of masculine traits in the context of help-seeking (eg, responsibility and strength). To inform the content development of the website, 14 qualitative problem-centered interviews [25] were conducted with men who had personal experiences of suicidal thoughts and behaviors and who had each attempted suicide at least once. The participants were between 19 and 61 years old. The interviews focused on various topics, including the development or signs of suicidal behavior, problem-solving behavior, communication of suicidal intent, support systems, protective factors, stigmatization of suicidal behavior, and possibilities for suicide prevention. The data were analyzed using structured qualitative content analysis [26]. The findings of Spahn et al [27] on facilitating and hindering aspects of help-seeking among men with suicidal behavior were directly incorporated into the development of the website’s content. In line with a participatory approach, the website content was also reviewed not only by experts (including therapists) but also by 3 men with lived experiences of suicidality. Their feedback was incorporated, and the content was further refined in a joint iterative process.

The primary goal of the website [17] is to encourage men at risk of suicide to seek help, but it also aims to inform and reduce stigma. The website addresses and supports various steps of the help-seeking process [28]: recognizing the problem, assessing one’s own situation (eg, subpages “Warning Signs” or “Assessing Your Own Situation”), deciding to seek help and overcoming barriers to help-seeking (eg, subpages or films on fear of stigma, fear of [involuntary] hospitalization, lack of trust in help services), and finding and accessing help (eg, the subpage “Navigating the Support System,” communicating suicidal intentions).

**Figure 1.**
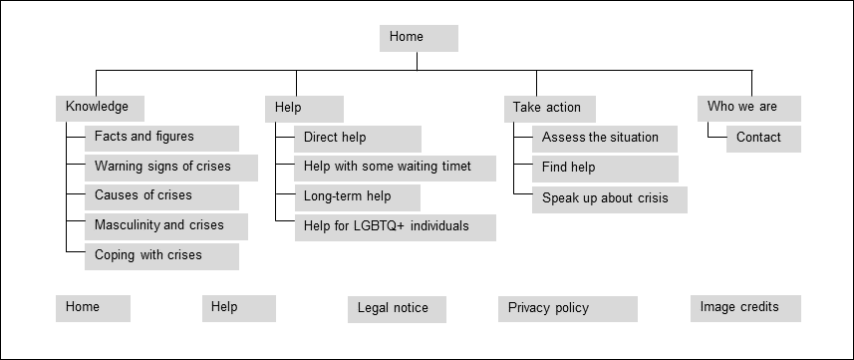
Site map of the website [17]. LGBTQ+: lesbian, gay, bisexual, transgender/transsexual, queer, and other minority sexual orientations and gender identities.

**Figure 2.**
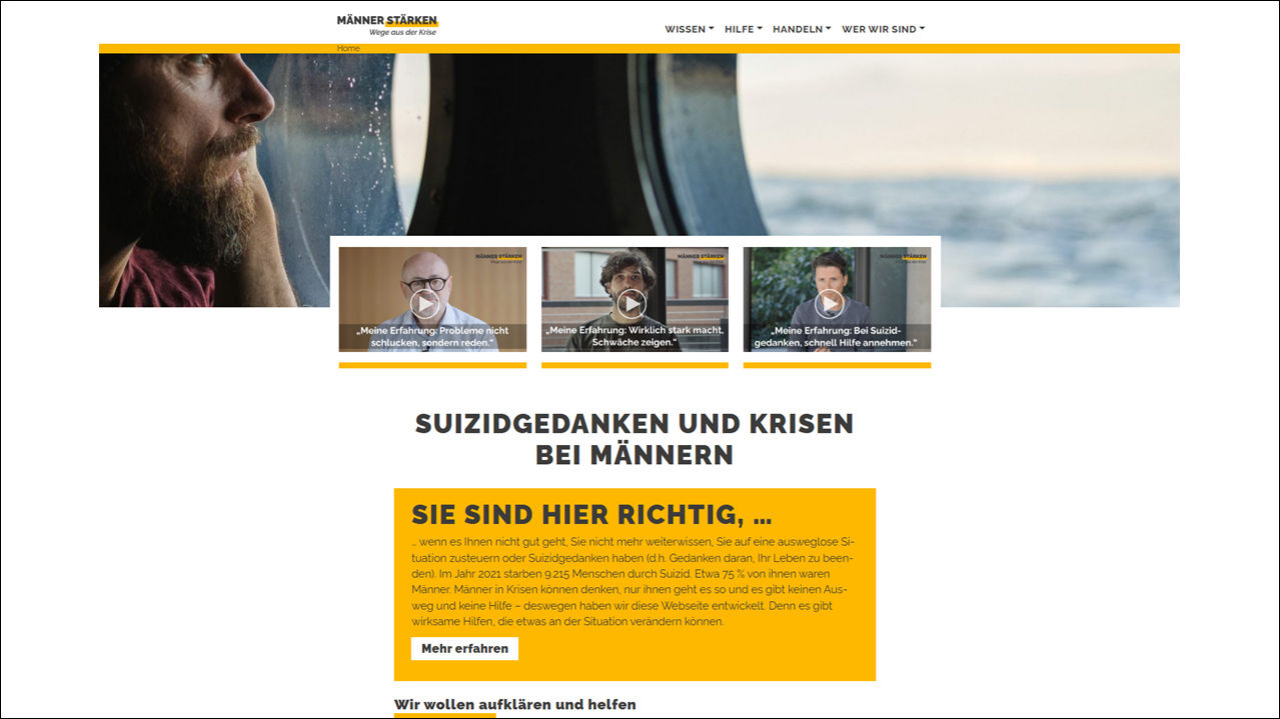
Homepage of the website [17].

Figure 1 illustrates the website’s structure and provides an overview of the content presented on its subpages. Figure 2 shows the landing page of the website. The website provides content in the form of texts, graphics, and short films. The homepage features 3 main short films portraying 3 male protagonists: 1 younger, 1 middle-aged, and 1 older. The subpages include additional short films in which the 3 protagonists talk about specific topics, such as masculinity norms and seeking help. In the short films on the homepage, men talk about their own experiences with mental health crises, suicidal ideation, seeking help, and recovery. Studies have shown that positive narratives about coping with suicidal ideation can reduce suicidal thoughts (“Papageno effect”) [29]. Films can also have a destigmatizing effect. However, the findings regarding changes in attitudes toward help-seeking remain inconclusive [15,30-32].

This study examines the extent to which the website and its content achieved its goals based on three research questions:

1. How is the website accessed (number of visits and page views)?
2. Is the site perceived as helpful, and can it encourage men to seek help?
3. Does a short film on suicide prevention increase the intention to seek help?

## Methods

The study combines quantitative web analytics and survey data with a qualitative analysis of open-ended responses from a feedback form to address the above questions.

### Ethical Considerations

Ethical approval was granted by the ethics committee of the Medical Faculty of Leipzig University (365/21-ek). Participants in the short film study were informed at the outset about the study content, procedures, voluntariness, and data protection, and provided informed consent. Participants received compensation of €8.00 for their participation. The individuals appearing in the videos on the website have given their consent for the videos to be published.

### Website Access

Matomo [33], a web behavior analysis tool, was installed to study the website usage by collecting data on user behavior. Specifically, it records, among other things, when a visitor accesses the site, which subpages are visited, how much time is spent on each subpage, and from which subpage the visitor exits the website. For both practical use and data protection reasons, it was important to ensure the anonymity of website visitors. Therefore, Matomo was used as the data collection method, as it prevents the unique identification of individuals and removes the requirement for a cookie banner. This study examines the number of visits and how visitors used individual subpages from March 1, 2023, to December 31, 2024.

### Feedback on Helpfulness and Encouragement of Help-Seeking

The website includes a feedback form on 13 out of 21 subpages. The form contains the following introductory text: “Please answer the following questions. Your response will help us further develop this website. Your responses will be stored anonymously. Thank you!”. This is followed by 3 questions (“Is this website helpful to you?”, “Can this website motivate you to use support services?”, and “Do you need further information? If yes, what kind?”), each of which can be answered with “Yes,” “No,” or “Maybe.” After the last question, there is a free-text field with the note “Add ideas.”

This study analyzes feedback received via the form from March 1, 2023, to December 31, 2024. The responses to the 3 questions were analyzed quantitatively, and the responses from the free-text field were evaluated using thematic analysis [34]. Thematic analysis is a method for identifying, analyzing, and reporting patterns (themes) within qualitative data. As a first step, text segments containing substantive feedback on the website were coded. MAXQDA [35] was used for the coding process, and initial coding was conducted by CS. Subsequently, the coded texts and codes were discussed and refined through peer debriefing with HG and LS, as well as with other colleagues experienced in qualitative research. Then, they were clustered into higher-order themes. These were elaborated and substantiated based on the underlying text segments.

### Change in the Intention to Seek Help

The 3 short films are documentary-style interviews without a visible or audible interviewer (“talking head”). They are testimonies. Some men describe their experiences, while other experiential accounts are fictionalized narratives drawing on experiences reported by men in interviews [27]. The protagonists are either actors or men with lived experience. They included elements recommended by Sagar-Ouriaghli et al [24] for interventions in men’s suicide prevention. One of these 3 short films, featuring a young male protagonist (approximately 25‐30 years old), was tested in an online survey to determine its effect on the male participants’ intention to seek help.

The short film, lasting 6 minutes and 47 seconds, features an actor portraying a man who recounts how high-performance pressure and overwhelming work demands led to social withdrawal and depressive symptoms. After a breakup with his partner, he attempted suicide. He then describes seeking professional help and engaging in a recovery process marked by experiences of empathy, the rediscovery of personal strengths, and the development of new coping strategies. His narrative is accompanied by brief informational inserts on male suicidality and help-seeking behavior. The film concludes with contact details for professional support services.

G*Power software (version 3.1 [36]) was used to calculate the required sample size for the online survey seeking feedback on the film. Based on previous findings, we expected a moderate effect of the film on help-seeking intentions [37,38]. The calculation was conducted for a repeated-measures ANOVA using an effect size *d*. A correlation of 0.5 between the pre-, post-, and follow-up measurements was assumed. Statistical power was set at 0.95, and the significance level at .05. To detect the hypothesized effect, an initial sample of 43 participants was required. Considering an expected dropout rate of 25%, we aimed to recruit at least 54 participants.

Male-identified participants were recruited among students and academic networks at the University of Magdeburg between June and August 2023. A total of 64 people started the online survey on their intention to seek help. Of these, 57 completed the first phase. In the follow-up survey, 47 participants took part and were therefore included in the data analysis. Another 7 individuals were excluded from the analysis because the recorded time spent watching the short film indicated that they had not watched it, or because they did not identify as male.

The study was conducted using the online tool Social Science Survey [39]. Sociodemographic data were collected from the participants who also answered the General Help-Seeking Questionnaire (GHSQ [40,41]) before watching the short film, immediately afterward, and again 14 days later.

The 20-item GHSQ assesses self-reported intentions to seek help. It includes two 10-item scales addressing personal or emotional problems and suicidal ideation. Eight of the 20 items refer to private sources of help and 6 to professional sources. Thus, across problem types (personal or emotional problems, suicidal ideation), two subscales can be constructed for private and professional sources of help. The remaining items cannot be clearly assigned to either category (eg, “spiritual support”). Participants rated the likelihood of seeking help (eg, from a friend, doctor, or psychologist) on a 7-point Likert scale (1=extremely unlikely to 7=extremely likely). Item 10 (free-text) was excluded. Mean scores were calculated for each scale, with higher scores indicating stronger help-seeking intentions. The GHSQ demonstrates good psychometric properties. Internal consistency and test-retest reliability for the 2 problem scales were acceptable (*α*=.83/.70; *r*=0.88/0.86 [41]). Convergent validity has been shown with actual help-seeking and perceived quality of prior care. In our sample, internal consistency was *α*=.73 (suicidal ideation), *α*=.76 (private), and *α*=.79 (professional). The GHSQ was originally developed in English and was therefore translated for the German-speaking participants using a forward-backward translation procedure.

Before watching the film, the participants completed the Suicidal Ideation and Behavior Scale (SIBS [42]) and the 9-item Patient Health Questionnaire (PHQ-9 [43]). The SIBS comprises 6 items assessing the frequency of suicidal thoughts, intentions, reparation, and planning, rated from 1 (never) to 6 (many times every day). Two additional dichotomous (yes or no) items assess suicide attempts in the past 4 weeks and lifetime. One item on the frequency of past attempts was excluded. A sum score was calculated from items 1‐6; suicide attempt items were reported separately. The SIBS shows excellent internal consistency (*α*=.92) and strong test-retest reliability (*r*=0.83) [42]. In our sample, internal consistency was good (*α*=.77). The PHQ-9 is a 9-item self-report instrument for screening and assessing depressive symptoms over the past 2 weeks. The validated German version was used [44]. Items (eg, “little interest or pleasure in doing things”) are rated on a 4-point scale from 1 (not at all) to 4 (nearly every day). Total scores above 9 indicate minor and above 14 moderate-to-severe depression. The PHQ-9 is a reliable and valid instrument [43,44], with good internal consistency (*α*=.88). In our sample, internal consistency was *α*=.83.

Data analyses were conducted with the software R (version 4.3.0; R Core Team) [45] in the RStudio environment using the R package “rstatix” (version 0.7.2 [46]). To test for significant differences between measurement time points, a 1-way repeated-measures ANOVA was performed for each target variable. As a total of 4 tests were carried out, a Bonferroni correction was made by multiplying the *P* values by the number of comparisons. The reported *P* values reflect this adjustment. Prior to the analysis, the model assumptions for each ANOVA were checked. Post hoc analyses were performed using 2-tailed paired *t* tests comparing the 3 time points pairwise; here, too, *P* values were adjusted with the Bonferroni correction.

## Results

### Website Access

A total of 29,279 unique visits (single user session) were registered on the website via the Matomo analysis tool. Due to the degree of anonymization of visitors in the analysis tool (shortening of the IP address by the last 3 digits), it is not possible to determine how many visitors accessed the website repeatedly. For this reason, the number of visits is provided here. A visit may also involve viewing multiple subpages. The visits lasted an average of 1 minute and 35 seconds. On average, 2.1 actions were performed per visit on the website (page views, downloads, outbound links, and internal searches). Table 1 shows the most frequently visited subpages; the average time spent on the respective subpages; the bounce rate, which describes the proportion of visits that included only that subpage (without visiting any other subpages before or after); and the exit rate. This describes how often the subpage was the last subpage visited on the website.

**Table 1.** Key metrics on website visit behavior.

Page title	Page views	Bounce rate (%)	Average time on page (h:min:s)	Exit rate (%)
Home	31.019	73	00:00:35	88
Help	3.077	68	00:00:46	29
Help—direct help	1.993	80	00:01:08	49
Knowledge—warning signs of crisis	1.375	77	00:01:10	42
Knowledge—masculinity and crisis	1.233	76	00:00:47	47
Help—help with some waiting time	1.047	79	00:01:11	45

### Feedback on Helpfulness and Encouragement of Help-Seeking

During the study period, a total of 291 completed feedback forms were analyzed. There were no indications that any of these forms were completed for advertising purposes or any similar purpose. As the feedback form was completed anonymously, it is not possible to provide any information about the individuals who completed it. However, the thematic feedback in the free-text field allows cautious conclusions to be drawn about the respondents. The feedback was analyzed using quantitative descriptive statistics and qualitative thematic analysis. The number of yes-no-maybe responses to the 3 questions is shown in Table 2.

**Table 2.** Results of the feedback form on the website (N=291).

Response	1. “Is this website helpful to you?” n (%)	2. “Can this website motivate you to use support services?” n (%)	3. “Do you need further information?” n (%)
Yes	201 (69.1)	174 (59.8)	85 (29.2)
No	42 (14.4)	42 (14.4)	109 (37.5)
Maybe	48 (16.5)	75 (25.8)	97 (33.3)

A total of 69.1% (n=201) report that the website was helpful to them, and 59.8% (n=174) confirm that the website could motivate them to seek help. In total, 14.4% (n=42) answered “no” to both questions. The free-text field after the last question was used by 75 (25.8%) of the respondents to provide thematic feedback. Of these 75 thematic responses, 57 (76%) referred to a positive assessment of the website (answering either “yes” or “maybe” to questions 1 and 2) and 18 (24%) referred to a negative assessment of the website (answering “no” to questions 1 and/or 2). This means that 42.9% of the negative responses to questions 1 and/or 2 also provided thematic feedback. The thematic analysis of the feedback indicates that it was primarily provided by men affected by suicidality, relatives, and professionals working in psychosocial care. To maintain clarity, only an overview of the themes identified through the thematic analysis is presented below. The positive feedback (at least one of the first two questions was answered affirmatively; n=38, 50.7%) relates in particular to the importance and need for suicide prevention services specifically aimed at men. This feedback was provided by affected individuals, as well as by relatives and professionals.

One example reads:

I think the site is great and highly relevant. From my own experience, I know how long it can take to admit that you need help. It is incredibly important that men, too, dare to talk more about depression and suicidal thoughts...Thank you for the website and your work!...

In addition, the following themes were identified in the data: description of personal experiences, offer to collaborate, provision of information on support services, expressions of gratitude, needs of relatives and professionals, lack of specialized support services, technical issues, and the expressed desire for more experiential reports. Example texts include: “I am experiencing a professional crisis. I have now sought help and hope to overcome this crisis soon” (description of personal experience) or “As an occupational therapist, I would need informational materials to display in the waiting room” (needs of professionals).

The focus of the negative feedback (at least one of the first 2 questions was answered negatively; n=18, 24%) was on anger and frustration or despair that individuals experience in connection with the psychosocial support system recommended on the website. In their feedback, respondents reported having negative experiences with the support system or described situations in which they did not receive support from the system despite being in need.

This is illustrated by the following example:

I’ve already tried all the support services you mentioned, but unfortunately none of them provided any relief. Sometimes I even felt stigmatized or retraumatized by therapists....The support services you mentioned are just the same old system, a revolving door that people keep ending up in...

Some negative responses reflected differing expectations and needs among users regarding the website. While some found the amount of information overwhelming, others expressed a desire for more detailed content. The following themes were identified in the free-text responses: frustration regarding gender-inclusive language, no new information provided, unclear purpose, technical issues or requests for changes, lack of specialized support services or difficulty locating support services, and the desire for more experiential reports. Examples of text passages include the following: “Your support services do not provide any new information” (no new information) or “Please stop with the gender-inclusive language!” (frustration regarding gender-inclusive language).

### Change in the Intention to Seek Help

Table 3 presents the sociodemographic characteristics of the sample (n=40). The sample is relatively young (mean age 24.53, SD 5.03 y) and shows elevated depression scores (PHQ-9), and more than one third report prior experiences with suicidal thoughts and behaviors.

**Table 3.** Descriptive statistics of sample characteristics (n=40).

Variable	Respondents
Age (y), mean (SD; range)	24.53 (5.03; 18-44)
Gender (free-text gender identity or assigned gender at birth), n (%)	
Cis male (male, men, cis male/male)	35 (87.5)
Trans-masculine (male, trans-masculine or female)	3 (7.5)
Other (not provided, “Doesn’t Matter”/male)	2 (5)
Education, n (%)	
Still in school	1 (2.5)
Finished secondary school	3 (7.5)
Qualification for university entrance	28 (70.0)
University degree	8 (20.0)
Occupation, n (%)	
Secondary school student	1 (2.5)
Apprentice	2 (5)
University student	34 (85)
Employee	3 (7.5)
Participants with elevated PHQ-9^a^ scores, n (%)	
Moderate depression (PHQ-9=10‐14)	9 (22.5)
Moderate severe depression (PHQ-9=15‐19)	3 (7.5)
PHQ-9 score, mean (SD)	7.36 (4.43)
Participants who attempted suicide, n (%)	
In the last 4 weeks	0 (0)
Lifetime	5 (12.5)
Participants with SIBS^b^ score >0, n (%)	14 (35)

a PHQ-9: 9-item Patient Health Questionnaire.

b SIBS: Suicidal Ideation and Behavior Scale.

**Figure 3.**
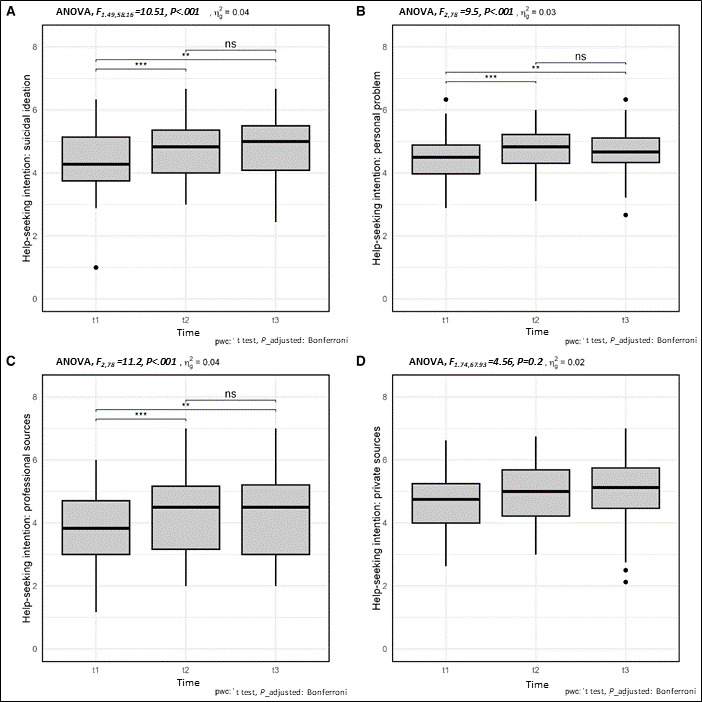
Boxplots of the intention to seek help (General Help-Seeking Questionnaire [GHSQ] scores) by measurement time points. ns: nonsignificant. **P<.01, ***P<.001.

The intention to seek help, in the case of suicidal ideation (*F*_1.49,58.16_=10.51; *P*_adjusted=.002) as well as in the case of a personal problem (*F*_2,78_=9.50; *P*_adjusted=.001), increased significantly. This significant increase in the intention to seek help was also evident at follow-up. The same applies to the intention to seek professional help (*F*_2,78_=11.20; *P*_adjusted<.001). There was no significant increase in the intention to seek help from private sources directly after watching the film or at follow-up (*F*_1.74,67.93_=4.56; *P*_adjusted=.72). The exact data of the analyses can be found in Figure 3 and Table 4.

**Table 4.** Post hoc tests for intention to seek help before and immediately after watching the film as well as 2 weeks later.

Outcome		Paired *2-*tailed *t* test (t_1_-t_2_)				Paired 2-tailed *t* test (t_1_-t_3_)			
	Mean_t1_ (SD)	Mean_t2_ (SD)	Mean_diff_	*P*_adjusted	*d*	Mean_t3_ (SD)	Mean_diff_	*P*_adjusted	*d*
GHSQ^a^									
Suicidal ideation	4.34 (1.03)	4.75 (0.93)	0.41	<.001	0.67	4.80 (1.03)	0.46	.006	0.52
Personal problem	4.42 (0.70)	4.70 (0.76)	0.28	<.001	0.71	4.72 (0.84)	0.30	.005	0.54
Professional subscale	3.82 (1.12)	4.30 (1.28)	0.48	<.001	0.72	4.36 (1.34)	0.54	.002	0.59
Private subscale	4.66 (0.89)	4.95 (1.01)	0.29	.72	—^b^	4.95 (1.21)	0.29	—	—

a GHSQ: General Help-Seeking Questionnaire.

b Not applicable.

## Discussion

### Principal Findings

Online services are extremely important in suicide prevention, especially for target groups that are hard to reach, like men [47-49]. Websites can be accessed anonymously, from any location and at any time. The website [17] is the first suicide prevention website for men in Germany. It was developed with consideration of the limited research on how to effectively address men in online settings to prevent suicide. In line with a participatory approach, men with lived experience were actively involved in the development process. This exploratory study combines quantitative web analytics and survey data with a qualitative analysis of free-text responses to examine the extent to which the website achieves its primary objectives: encouraging men at risk of suicide to seek help, providing information, and reducing stigma. This study builds upon the investigation by Reifegerste et al [22], who demonstrated through qualitative interviews that the videos on the website [17] were perceived by men as engaging, clear, and empathetic. However, they were also considered too lengthy for a brief introduction. The balance between emotional and informational content was regarded as appropriate and supportive. Furthermore, the health information provided was viewed as credible and trustworthy.

In the period under review, the website received almost 30,000 visits. The information from the feedback form indicates that the website’s target groups (affected men, relatives, and professionals working in psychosocial care) were reached. The number of visits is difficult to interpret, as very few studies report usage figures for prevention websites [50], and therefore, no comparative data are available. Moreover, no conclusions can be drawn about the number of unique visitors based on the number of visits. Due to data protection requirements and the protection of anonymity, individual visitors cannot be identified using the web analytics data. If it is assumed that some visitors accessed the website multiple times, the number of unique visitors is likely to be lower than 30,000. An analysis of the thematic subpages accessed shows that the help pages (short-term and medium-term support) were visited most frequently, as well as pages addressing gender-specific topics (warning signs in men and masculinity in the context of crises).

The 291 responses submitted via the form on the website show that the majority of those who completed the form rated the service as helpful, encouraging them to seek help. The thematic feedback clearly emphasizes the high demand for suicide prevention services specifically tailored to men, while also highlighting the lack of such services to date. In addition to the positive feedback, 15% (n=42) of the respondents indicated that they did not find the website helpful and did not feel motivated to seek help. This substantial proportion may reflect several factors. Certainly, a website that primarily serves as a first point of contact and provides only information, insights into others’ experiences, and links to support services may not be the right resource for some men in (suicidal) crises. These individuals may require other forms of support, counseling, or therapy, which can be tailored to their individual needs. It should also be considered that the website targets a highly heterogeneous group—“men”—without differentiating between specific subgroups, such as age groups, minority groups, or other demographic characteristics. Consequently, many diverse perspectives and needs may not be directly addressed on the website and are instead generalized for the “majority.” This could also contribute to some visitors feeling that the website does not adequately speak to them. In addition, the negative feedback provided through the feedback form points to issues of trust and frustration in dealing with the support system and its professionals, a concern also reflected in the literature [51]. Approximately 1 quarter (n=75, 25.8%) of the completed feedback forms contained entries in the free-text field. This high proportion of substantive contributions suggests that the website encouraged visitors to actively engage with the topic and share their perspectives. At the same time, it must be noted that, as described above, it is not possible to determine who completed the form or whether individuals may have submitted feedback more than once.

The investigation of the use of the website and the feedback via the form on the website are primarily descriptive in nature. On the one hand, they offer a high degree of realism (external validity), as they capture actual use and authentic feedback from real visitors. On the other hand, they do not take place under controlled conditions (low internal validity) and therefore do not permit reliable conclusions about the target group, impact, related factors, and so forth.

The study also examined the effect of a short suicide prevention film—also available on the website—on men’s intentions to seek help. The short film portrays a young man describing the positive outcomes of seeking help after a suicidal crisis. The results showed that immediately after watching the short film, the intention to seek help increased significantly, both for suicidal thoughts and for personal problems. The intention to seek help from professionals also increased. The short film on suicide prevention thus has the desired effect. It was also shown that this significant increase in the intention to seek help remained even 14 days later (follow-up). However, the study also shows that there was no increase in the intention to seek help from friends and family. Since the film explicitly focuses on professional support rather than help from friends and family, this effect appears consistent with the content of the film.

The results are in line with previous studies showing that media narratives about overcoming crises can positively influence help-seeking behavior [15,29,32,37,52]. In addition, our study contributes to the still limited but growing body of research indicating that gender-specific approaches—particularly those incorporating male role models and addressing gender-specific aspects of suicide—have the potential to increase men’s willingness to seek professional help [15]. Finally, it should be emphasized that this study measured only the intention to seek help, not actual help-seeking behavior. Although such intentions are regarded as reliable indicators of later behavior [41], the direct measurement of future help-seeking behavior was not part of the study design.

### Limitations

There are limitations to this study that need to be acknowledged. In particular, a potential selection bias may limit the generalizability of the findings and may also lead to an underestimation or overestimation of the effects. The sample for the film study was predominantly young, consisting mainly of university students (see Table 3). Studies indicate that age and education influence help-seeking intentions in the context of mental health crises in different ways. Zhao et al [53] demonstrated in their review that help-seeking intentions are relatively high among university students. Mackenzie et al [54] further found that older populations with higher levels of education exhibited stronger help-seeking intentions than younger individuals. In addition, the sample studied had a rather high load in terms of depression and suicidality, indicating that the topic of suicide prevention may be relevant to them. However, not all study participants exhibited such levels of burden, which may also have influenced the results. Stas et al [20] demonstrated that among men reporting mental health problems, a suicide prevention website led to increased openness to communication and emotional expression. Further studies should investigate whether the increase in help-seeking intentions can be observed independently of levels of psychological distress.

While even small increases (*d*=0.52-0.72; Table 4) in help-seeking intentions could be clinically and preventively significant, as they may lead to earlier engagement with professional support, the results must be interpreted with caution regarding their clinical and preventive relevance. On the one hand, no reliable cut-off exists to determine whether the intention to seek help is present or absent. On the other hand, the limitations described regarding the generalizability of the findings must also be taken into account. To confirm the effects found on the intention-enhancing effect of the film or to extend them to other films with a similar content structure, further large-scale studies with more diverse populations are required. In both the examination of the film and the analysis of the content-related feedback, the small sample sizes limit the generalizability of the findings. Another important limitation is the absence of a control group. Without a control group, causal conclusions cannot be drawn; the findings should be replicated in future randomized controlled trials.

### Conclusion

In summary, the findings of this study suggest that the website has been well received by the target group and is effective with respect to its stated objectives. These findings complement existing evidence by Till et al [55], which demonstrates knowledge gains through suicide prevention websites and suggests a reduction in suicidal ideation among vulnerable groups. The short film, in particular, may serve as an easily disseminated and cost-effective approach to suicide prevention in men. Potential applications extend beyond the website, for example, through high-reach social media channels. As the qualitative interview results by Reifegerste et al [22] have shown, shortened versions of the film could also be used in this context. However, to examine the effectiveness of these gender-specific, suicide-preventive media and website content more closely, considerably more research—particularly randomized controlled trials studies—is needed. The website and this study provide starting points for further research and campaigns that specifically address the high suicide risk among men. The high number of men who die by suicide each year worldwide underscores the urgent need for such approaches to gender-specific suicide prevention.

## Abbreviations

GHSQ: General Help-Seeking Questionnaire
PHQ-9: 9-item Patient Health Questionnaire
SIBS: Suicidal Ideation and Behavior Scale
